# Supplementary material for: Relative quantification of BCL2 mRNA for diagnostic usage needs stable uncontrolled genes as reference
Source: PLoS One. 2020 Aug 12;15(8):e0236338. doi: 10.1371/journal.pone.0236338 (PMC7423076; doi:10.1371/journal.pone.0236338)
Supplement: S1 Table — (DOCX) [file pone.0236338.s001.docx]

**S1 Table: List of reference genes in literature**

| **Sr no** | **Gene** | **No of articles** | **Pubmed ID (PMID) list** |
| --- | --- | --- | --- |
| 1 | GAPDH | 38 | 10894833:10985361:11086178:11442504:12017294:12031655:12040449:12063019:12468436:12682639:12843001:12866371:14686494:14748434:15096562:15293568:15467427:15945375:16644881:17904413:19327122:19861891:20100355:20119960:20186011:20813001:21862359:21986632:23990051:24423144:24750974:25364581:25696013:26674556:28544358:28732214:29484276:30800245 |
| 2 | ABL1 | 30 | 10834406:11114130:12040450:14562124:14686494:14748434:15229149:16242775:17299092:17660274:18696070:19055671:19861891:21072821:22299211:24173087:26079545:26674556:27109508:27565907:28735486:29689094:30617397:30701458:30897165:30941573:31531087:31578694:30897165 |
| 3 | B2M | 25 | 10850546:11122110:11479425:11896543:11986399:12682639:14562124:14738435:15284861:15334542:16085543:16242775:16945414:17299092:17904413:19861891:20813001:23318495:23990051:24173087:25696013:26196328:26674556:27543594:29273915 |
| 4 | GUSB | 23 | 14562124:17476280:17904413:18223279:18806751:19861891:20037605:20813001:21723805:22266405:23541592:23876601:24173087:25236575:25815362:25872147:26196328:26674556:27109508:30065619:30897165:31578694:30897165 |
| 5 | ACTB | 18 | 10679823:11238301:11333300:12682639:14686494:16178036:16930142:17251199:17703174:19165483:20813001:23328642:24223824:25696013:27650030:28843266:30874583: 17904413 |
| 6 | HMBS | 9 | 10673752:10834406:11479425:14506704:15284861:15746080:16044449: 20813001:25236575 |
| 7 | HPRT1 | 5 | 12682639:17904413:20813001:25696013:26674556 |
| 8 | TBP | 5 | 12682639:19861891:20813001:23876601:31578694 |
| 9 | 18SrRNA | 4 | 11455976:14748434:15945375:17904413 |
| 10 | ALB | 3 | 11122110:11333300:24158833 |
| 11 | G6PD | 3 | 12454744:23876601:31578694 |
| 12 | PPIA | 2 | 12411587:12682639 |
| 13 | PRKG1 | 2 | 12682639:19861891 |
| 14 | UBC | 2 | 20813001, 25696013 |
| 15 | RPLP0 | 2 | 29484276:11331038 |
| 16 | CD71 | 2 | 15945375:17904413 |
| 17 | BCR | 2 | 10834406:27109508 |
| 18 | RPL13A | 2 | 25696013:25929957 |
| 19 | RPL37A | 1 | 20947696 |
| 20 | UBQLN2 | 1 | 31108950 |
| 21 | NPM1 | 1 | 19055671 |
| 22 | RPLP2 | 1 | 17904413 |
| 23 | CYCS | 1 | 17904413 |
| 24 | STAT3 | 1 | 15945375 |
| 25 | YWHAZ | 1 | 25696013 |
| 26 | TERT | 1 | 18663754 |
| 27 | PGGT1B | 1 | 31108950 |
| 28 | CD20 | 1 | 12411587 |
| 29 | RPL30 | 1 | 30874583 |
| 30 | CD8A | 1 | 11122110 |
| 31 | PSMB6 | 1 | 31108950 |
| 32 | UQCR2 | 1 | 31108950 |
| 33 | KLF4 | 1 | 20119960 |
| 34 | SOX5 | 1 | 20119960 |
| 35 | PCNA | 1 | 15467427 |
| 36 | RARA | 1 | 10834406 |
| 37 | MRPL19 | 1 | 20813001 |
| 38 | PGK1 | 1 | 17904413 |
| 39 | SF3A1 | 1 | 17703174 |
| 40 | MTIF2 | 1 | 16644881 |
| 41 | PGM1 | 1 | 21079657 |
| 42 | KMT2C | 1 | 11718452 |
| 43 | PSMC4 | 1 | 26196328 |
