# Supplementary material for: Relative quantification of BCL2 mRNA for diagnostic usage needs stable uncontrolled genes as reference
Source: PLoS One. 2020 Aug 12;15(8):e0236338. doi: 10.1371/journal.pone.0236338 (PMC7423076; doi:10.1371/journal.pone.0236338)
Supplement: S2 Table — (DOCX) [file pone.0236338.s002.docx]

**S2 Table** List of BCL2 primers from literature

| SN | Amplicon length | Nucleotide region | Isoform | Reference |
| --- | --- | --- | --- | --- |
| 1 | 301 | 79-379 | Alpha & Beta | Elumalai, et.al (2012)^1^ |
| 2 | 148 | 191-338 | Alpha & Beta | Mansurabadi, et.al (2017)^2^ |
| 3 | 185 | 962-1146 | Alpha | Goff, et.al (2013)^3^ |
| 4 | 134 | 1011-1144 | Alpha | Stamati, et.al (2015)^4^ |
| 5 | 124 | 1018-1141 | Alpha | Xia, et.al (2015)^5^ |
| 6 | 357 | 1208-1564 | Alpha | Dai, et.al (2017)^6^ |
| 7 | 114 | 2714-2827 | Alpha | Liu, et.al (2005)^7^ |
| 8 | 94 | 3462-3555 | Alpha | Shen, et.al (2004)^8^ |
