# Supplementary material for: Relative quantification of BCL2 mRNA for diagnostic usage needs stable uncontrolled genes as reference
Source: PLoS One. 2020 Aug 12;15(8):e0236338. doi: 10.1371/journal.pone.0236338 (PMC7423076; doi:10.1371/journal.pone.0236338)
Supplement: S3 Table — (DOCX) [file pone.0236338.s003.docx]

**S3 Table.** List of unqualified primers

| Primer | Accession No | Sequence (5' - 3') | Amplicon Length (bp) | Tm (°C) |
| --- | --- | --- | --- | --- |
| TMEM187 | NM_003492 | Fw 5'-CTCACTCGTGAACATGGCCTA-3' | 101 | 61.5 |
|  |  | Rv 5'-CTGCGAACACGTCCTTCAG-3' |  | 60.7 |
| HAUS7 | NM_017518 | Fw 5'-AGCTCACTGAAAGGGGTCC-3' | 153 | 60.9 |
|  |  | Rv 5'-ATCGAGCAACTGGTCCATGAA-3' |  | 61.5 |
| MBLAC2 | NM_203406 | Fw 5'-CGCGGGGACAACTTTGAGA-3' | 161 | 62.2 |
|  |  | Rv 5'-GTGAGCTGTCTGTCACCAAGG-3' |  | 62.6 |
| F8 | NM_000132 | Fw 5'-GCATTCGCAGCACTCTTCG-3' | 160 | 61.8 |
|  |  | Rv 5'-GAGGTGAAGTCGAGCTTTTGAA-3' |  | 60.5 |
| TMEM116 | NM_001193453 | Fw 5'-TCATAAAGCTGACTAAGCCACAG-3' | 77 | 60 |
|  |  | Rv 5'-AGATGTTGCCGTTAGAGCCTG-3' |  | 62.1 |
| TTC30B | NM_152517.3 | Fw 5'-CTCTACAAGGAGGGACAGTAT-3' | 98 | 55.59 |
|  |  | Rv 5'-AAAGCCAGGTTGTAGGAAAG-3' |  | 55.54 |
| FGD1 | NM_004463 | Fw 5'-GAAGCTCGGAACCGCAGTT-3' | 80 | 62.6 |
|  |  | Rv 5'-TGGAAGCAATAGATGGAGCAGAT-3' |  | 61.2 |
| GLB1L | NM_024506 | Fw 5'-CCACAGCCTGGGGTCTATAAC-3' | 85 | 61.6 |
|  |  | Rv 5'-TGACCAACAGGTTCGCTAGAG-3' |  | 61.5 |
