# Supplementary material for: Relative quantification of BCL2 mRNA for diagnostic usage needs stable uncontrolled genes as reference
Source: PLoS One. 2020 Aug 12;15(8):e0236338. doi: 10.1371/journal.pone.0236338 (PMC7423076; doi:10.1371/journal.pone.0236338)
Supplement: S4 Table — (DOCX) [file pone.0236338.s004.docx]

**S4 Table.** Literature explaining analysis and selection of reference gene

| First Author (year) | Proposed Reference Genes | Tissue | Disease context | Selection method |
| --- | --- | --- | --- | --- |
| Villegas-Ruiz (2019)^9^ | PSMB6, PGGT1B, UBQLN2, UQCR2 | Bone marrow | ALL | Analysis of pan-cancer microarray data followed by ddPCR validation in ALL samples |
| Potashnikova (2015) ^10^ | YWHAZ, UBC, ACTB | Spleen, lymph nodes and peripheral blood | Multiple forms of Non-Hodgkin Lymphoma | qPCR validation and stability analysis using geNorm, NormFinder and BestKeeper |
| MacRae (2013)^11^ | HNRNPL, EIF4H, PSMA1 | Peripheral blood | Leukemia | Analysis of RNAseq data to rank genes by CV and MFC followed by qPCR validation |
| Valceckiene (2010)^12^ | B2M, HPRT1, GUSB | CD19+ cells isolated from peripheral blood | B-cell CLL | qPCR validation and stability analysis using geNorm, NormFinder and BestKeeper |
| Green (2009)^13^ | GUSB, TBP and ABL1 | Lymph node snap frozen and FFPE | DLBCL | qPCR validation and stability analysis using geNorm and NormFinder |
| Rulcova (2007)^14^ | B2M | Peripheral blood | CML (BCR-ABL+) | Detection of mRNA level by qPCR during course of therapy and analysis of variation |
| Lee (2006)^15^ | GUSB | Peripheral blood and bone marrow | CML (BCR-ABL+) | Detection of mRNA level by qPCR during course of therapy and analysis of variation |
| Wang (2006)^16^ | GUSB | Peripheral blood and bone marrow | CML (BCR-ABL+) | Detection of mRNA level by qPCR during course of therapy and during degradation and analysis of variation |
| Weisser (2004)^17^ | ABL | Peripheral blood | AML | Detection of mRNA level by qPCR over different transcript fusion genotypes and analysis of variation |
| Beillard (2003)^18^ | ABL | Peripheral blood, bone marrow and peripheral blood stem cells | ALL, AML, CML | Detection of mRNA level by qPCR over different patient groups and analysis of variation |
