# Supplementary material for: Relative quantification of BCL2 mRNA for diagnostic usage needs stable uncontrolled genes as reference
Source: PLoS One. 2020 Aug 12;15(8):e0236338. doi: 10.1371/journal.pone.0236338 (PMC7423076; doi:10.1371/journal.pone.0236338)
Supplement: S5 Table — (DOCX) [file pone.0236338.s005.docx]

**S5 Table.**  Z-score Medoid values

| **Cluster #** | **CV** | **MAD** | **1 - p** |
| --- | --- | --- | --- |
|  | **TCGA-LAML** | | |
| 1* | -0.621 | -0.757 | 0.233 |
| 2 | 0.573 | 0.957 | 0.233 |
|  | **TARGET-AML** | | |
| 1* | -0.583 | -0.638 | 0.058 |
| 2 | 0.509 | 1.004 | 0.058 |
|  | **GDC-DLBC** | | |
| 1* | -0.491 | -0.818 | 0.023 |
| 2 | 0.166 | 0.764 | 0.023 |
|  | **MMRF-MM** | | |
| 1 | 0.393 | 1.107 | 0.025 |
| 2* | -0.564 | -0.820 | 0.025 |

*Selected clusters
