# Supplementary material for: Relative quantification of BCL2 mRNA for diagnostic usage needs stable uncontrolled genes as reference
Source: PLoS One. 2020 Aug 12;15(8):e0236338. doi: 10.1371/journal.pone.0236338 (PMC7423076; doi:10.1371/journal.pone.0236338)
Supplement: S6 Table — (DOCX) [file pone.0236338.s006.docx]

**S6 Table** List of selected genes

| SN | HGNC symbol | Chromosome No | Band | Mean CV | Mean Euclidean Distance | Median Expression Quartile | CQR |
| --- | --- | --- | --- | --- | --- | --- | --- |
| 1 | FGD1 | X | p11.22 | 0.654 | 0.307 | (2,2,2,2) | 8 |
| 2 | GLB1L | 2 | q35 | 0.454 | 0.374 | (2,2,2,2) | 8 |
| 3 | TTC30B | 2 | q31.2 | 0.668 | 0.406 | (2,2,2,2) | 8 |
| 4 | NAT1 | 8 | p22 | 0.564 | 0.555 | (2,2,2,2) | 8 |
| 5 | F8 | X | q28 | 0.618 | 0.261 | (2,2,3,2) | 9 |
| 6 | TMEM116 | 12 | q24.13 | 0.464 | 0.389 | (2,2,2,3) | 9 |
| 7 | TSGA10 | 2 | q11.2 | 0.577 | 0.561 | (2,2,2,3) | 9 |
| 8 | NANP | 20 | p11.21 | 0.392 | 0.788 | (2,3,2,2) | 9 |
| 9 | HAUS7 | X | q28 | 0.566 | 0.036 | (2,2,3,3) | 10 |
| 10 | MBLAC2 | 5 | q14.3 | 0.512 | 0.204 | (3,2,2,3) | 10 |
| 11 | RIC8B | 12 | q23.3 | 0.476 | 0.285 | (3,2,3,2) | 10 |
| 12 | FBXW9 | 19 | p13.13 | 0.577 | 0.315 | (3,3,2,2) | 10 |
| 13 | PPP1R3B | 8 | p23.1 | 0.586 | 0.327 | (2,3,3,2) | 10 |
| 14 | PLEKHM3 | 2 | q33.3 | 0.477 | 0.349 | (3,3,2,2) | 10 |
| 15 | TMEM187 | X | q28 | 0.577 | 0.453 | (2,3,2,3) | 10 |
| 16 | ANKRD26 | 10 | p12.1 | 0.502 | 0.600 | (3,2,3,2) | 10 |
| 17 | ACY1 | 3 | p21.2 | 0.658 | 0.653 | (2,2,3,3) | 10 |
| 18 | PTCD2 | 5 | q13.2 | 0.434 | 0.679 | (3,2,2,3) | 10 |
| 19 | JMJD4 | 1 | q42.13 | 0.512 | 0.757 | (2,2,3,3) | 10 |
