# Supplementary material for: Relative quantification of BCL2 mRNA for diagnostic usage needs stable uncontrolled genes as reference
Source: PLoS One. 2020 Aug 12;15(8):e0236338. doi: 10.1371/journal.pone.0236338 (PMC7423076; doi:10.1371/journal.pone.0236338)
Supplement: S7 Table — (DOCX) [file pone.0236338.s007.docx]

**S7 Table.** Individual and combined stability rank and scores of candidate reference genes

| Gene | geNorm | | NormFinder | | BestKeeper | | Reffinder | | Combined rank |
| --- | --- | --- | --- | --- | --- | --- | --- | --- | --- |
|  | **Rank** | **Score (M)** | **Rank** | **Score** | **Rank** | **Score (r)** | **Rank** | **Score (geo mean)** |  |
| PTCD2 | 1 | 1.689 | 2 | 0.62 | 1 | 0.860 | 1 | 1.78 | 1.19 |
| PPP1R3B | 1 | 1.689 | 1 | 0.57 | 7 | 0.775 | 4 | 4.79 | 2.30 |
| FBXW9 | 3 | 1.869 | 3 | 0.62 | 4 | 0.818 | 3 | 2.45 | 3.22 |
| NANP | 5 | 2.379 | 8 | 0.92 | 2 | 0.840 | 2 | 2.34 | 3.56 |
| RIC8B | 6 | 2.608 | 5 | 0.7 | 3 | 0.819 | 5 | 4.90 | 4.61 |
| JMJD4 | 4 | 2.019 | 6 | 0.71 | 6 | 0.814 | 8 | 6.65 | 5.83 |
| PLEKHM3 | 7 | 2.716 | 7 | 0.75 | 5 | 0.816 | 9 | 6.70 | 6.85 |
| NAT1 | 8 | 2.803 | 4 | 0.65 | 8 | 0.772 | 10 | 8.24 | 7.11 |
| ANKRD26 | 9 | 2.874 | 9 | 1 | 9 | 0.458 | 7 | 6.18 | 8.45 |
| TSGA10 | 10 | 2.986 | 10 | 1.37 | 10 | 0.457 | 6 | 5.62 | 8.80 |
