# Supplementary material for: Relative quantification of BCL2 mRNA for diagnostic usage needs stable uncontrolled genes as reference
Source: PLoS One. 2020 Aug 12;15(8):e0236338. doi: 10.1371/journal.pone.0236338 (PMC7423076; doi:10.1371/journal.pone.0236338)
Supplement: S8 Table — (DOCX) [file pone.0236338.s008.docx]

**S8 Table.** Relative expression of GAPDH and the proposed normalization factor

| **PATIENT_ID** | **Proposed Normalization Factor** | **GAPDH** |
| --- | --- | --- |
| L105 | -9.778 | -11.442 |
| L106 | -9.251 | -8.422 |
| L117 | 2.888 | 3.180 |
| L120 | 1.155 | -6.591 |
| L130 | 2.732 | -0.375 |
| L143 | -1.623 | -6.584 |
| L145 | -1.484 | -3.980 |
| L148 | 0.628 | -3.501 |
| L151 | 2.812 | 1.380 |
| L156 | 2.717 | -2.094 |
| L158 | 4.835 | 3.643 |
| L160 | 0.919 | 3.787 |
| L174 | 0.329 | -13.243 |
| L180 | 1.641 | -8.524 |
| L192 | -3.134 | -10.161 |
| L194 | -1.966 | -6.839 |
| L196 | 0.447 | -9.773 |
| L199 | -2.073 | -9.319 |
| L200 | -2.611 | -8.206 |
| L203 | -5.674 | 0.248 |
| L204 | 7.051 | -4.938 |
| L205 | 8.269 | -6.360 |
| L207 | -3.488 | -11.222 |
| L210 | 7.936 | -5.600 |
| L212 | 0.198 | -1.712 |
| L214 | -6.913 | -2.129 |
| L215 | 0.919 | -4.224 |
| L216 | 0.460 | 0.884 |
| L218 | 0.871 | -0.559 |
| L219 | 1.841 | -6.068 |
| L220 | 7.961 | 8.134 |
| L221 | 4.285 | -7.072 |
| L224 | -0.756 | -3.611 |
| L250 | -0.205 | -2.563 |
| L254 | 1.384 | -4.182 |
