# Supplementary material for: Relative quantification of BCL2 mRNA for diagnostic usage needs stable uncontrolled genes as reference
Source: PLoS One. 2020 Aug 12;15(8):e0236338. doi: 10.1371/journal.pone.0236338 (PMC7423076; doi:10.1371/journal.pone.0236338)
Supplement: S9 Table — (DOCX) [file pone.0236338.s009.docx]

**S9 Table**. Sequence similarity and identity with corresponding genes in mice, rat and guinea pig

|  | **Model Organism*** | | | | | |
| --- | --- | --- | --- | --- | --- | --- |
|  | Mouse,  *Mus musculus* | | Rat,  *Rattus norvegicus* | | Guinea Pig,  *Cavia porcellus* | |
| **Gene** | **Identity** | **Similarity** | **Identity** | **Similarity** | **Identity** | **Similarity** |
| **PTCD2** | 72.40% | 85.80% | 72.40% | 85.20% | 74.00% | 86.30% |
| **PPP1R3B** | 89.50% | 94.40% | 89.50% | 94.70% | 89.80% | 94.40% |
| **FBXW9** | 72.70% | 80.90% | 73.00% | 81.80% | 72.70% | 80.10% |

*All values are results of alignments performed with respective human genes
