# Supplementary figures and images for: Relative quantification of BCL2 mRNA for diagnostic usage needs stable uncontrolled genes as reference
Source: PLoS One. 2020 Aug 12;15(8):e0236338. doi: 10.1371/journal.pone.0236338 (PMC7423076; doi:10.1371/journal.pone.0236338)

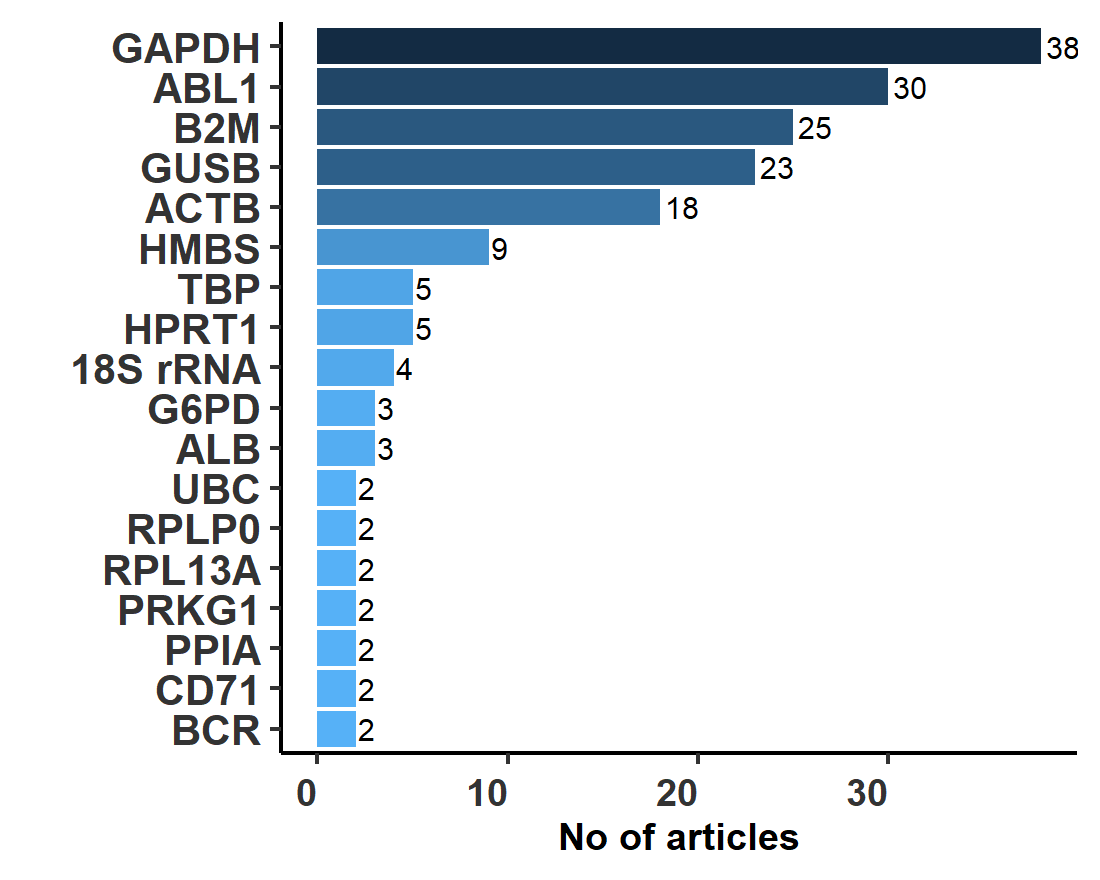

Supplement: S1 Fig — (TIFF) [file pone.0236338.s010.tiff]

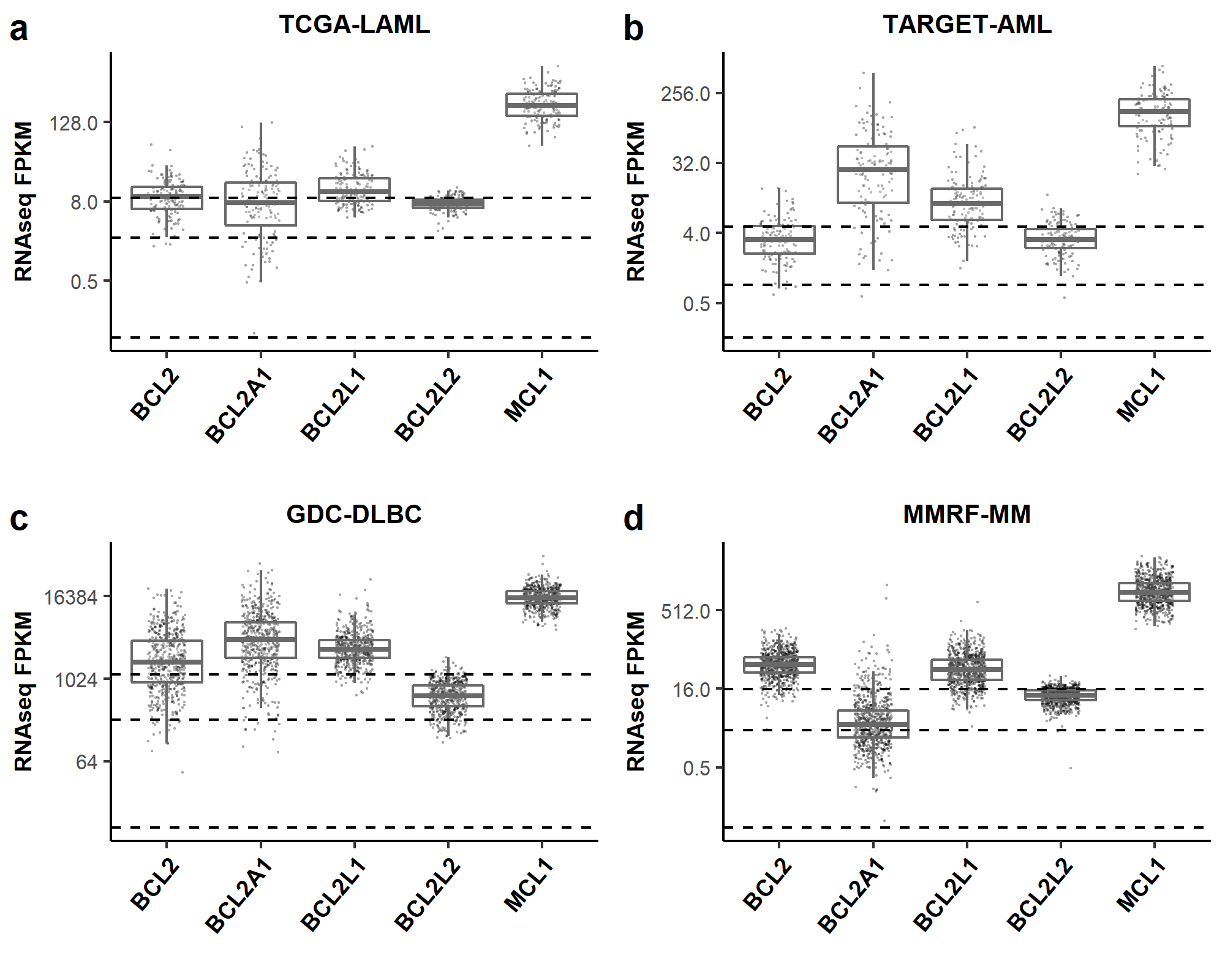

Supplement: S2 Fig — (TIFF) [file pone.0236338.s011.tiff]

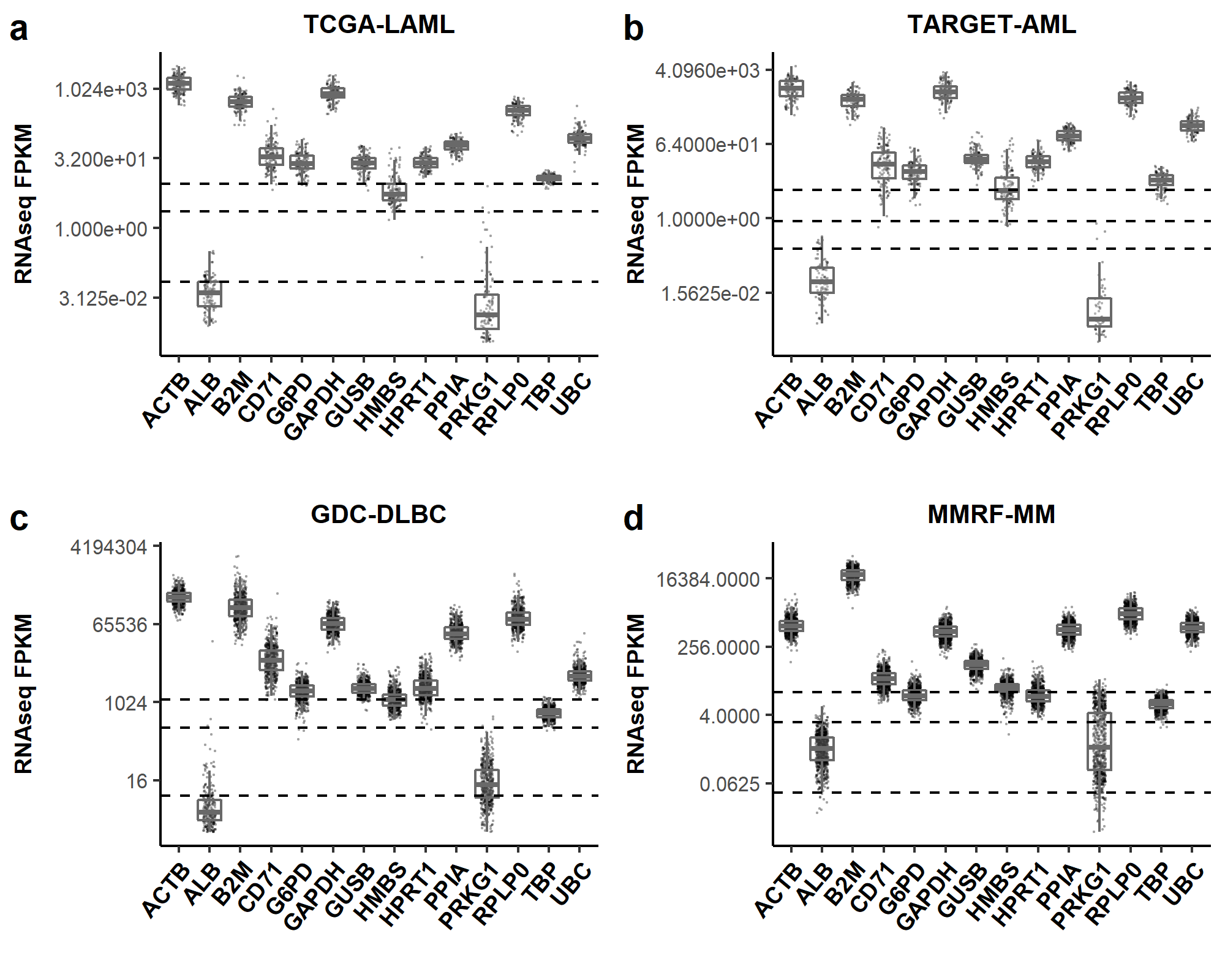

Supplement: S3 Fig — (TIFF) [file pone.0236338.s012.tiff]

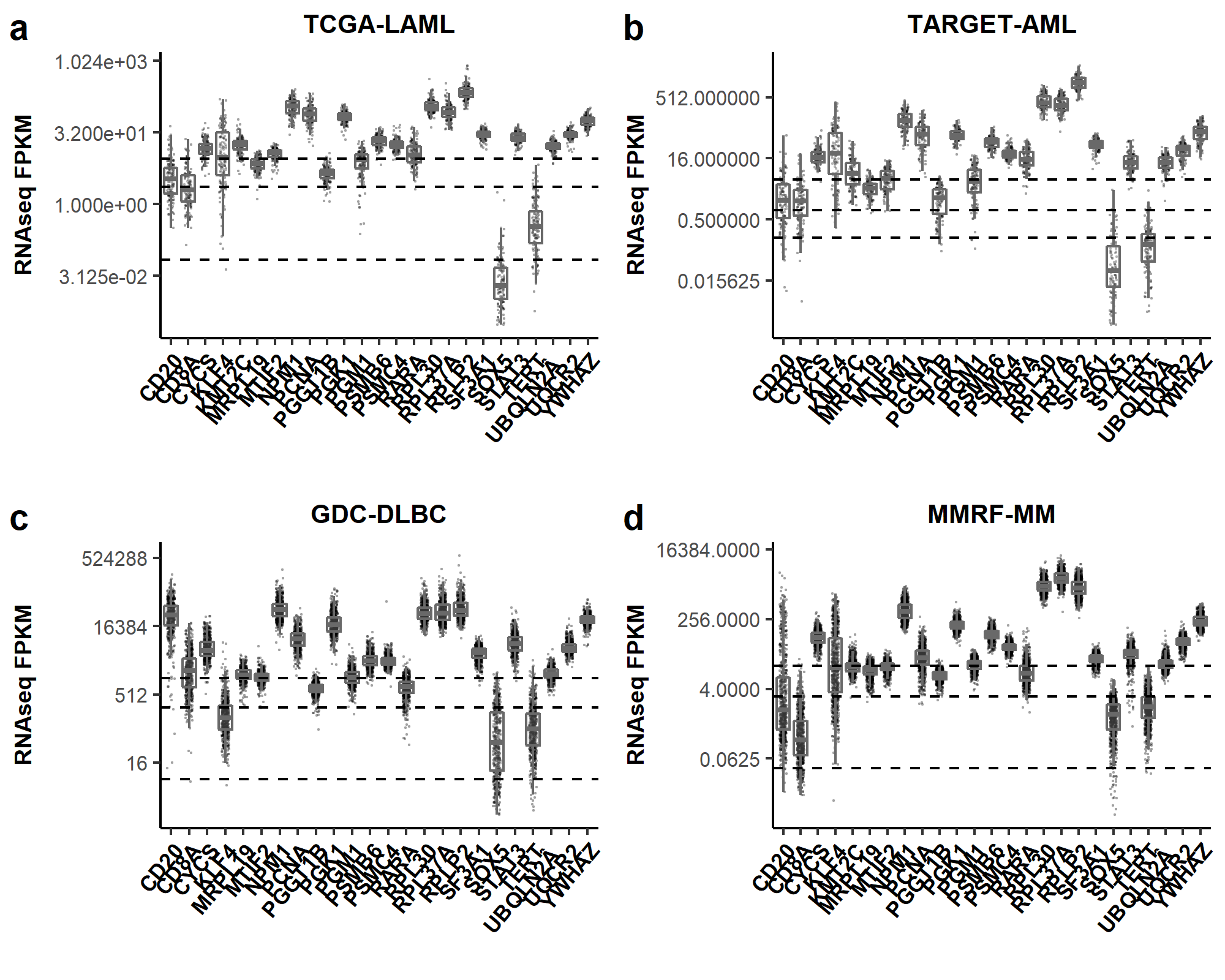

Supplement: S4 Fig — (TIFF) [file pone.0236338.s013.tiff]

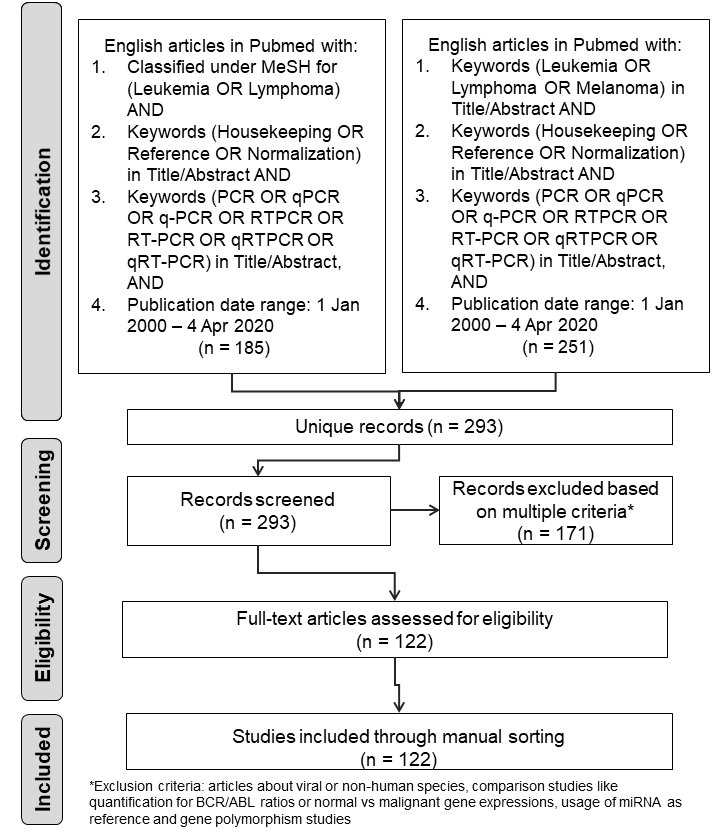

Supplement: S5 Fig — (TIFF) [file pone.0236338.s014.tiff]

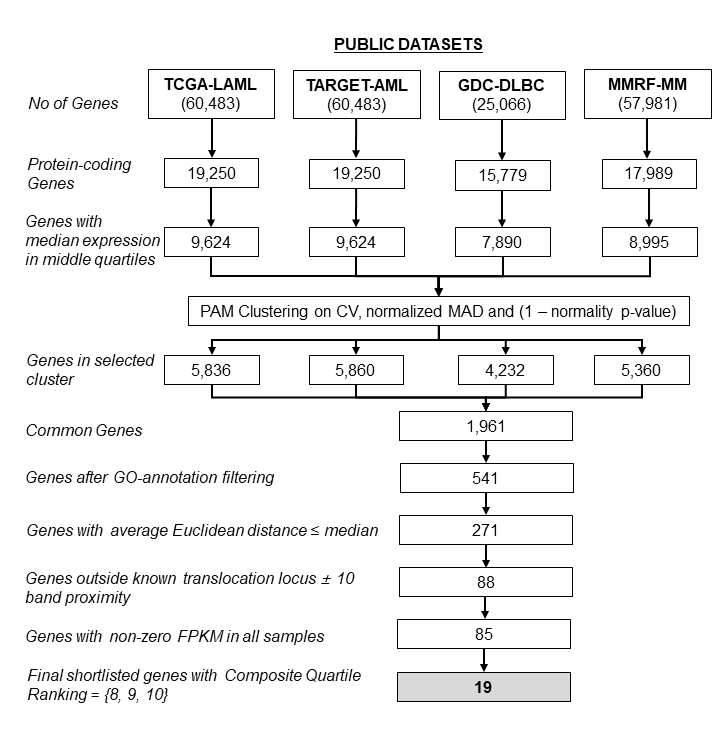

Supplement: S6 Fig — (TIFF) [file pone.0236338.s015.tiff]

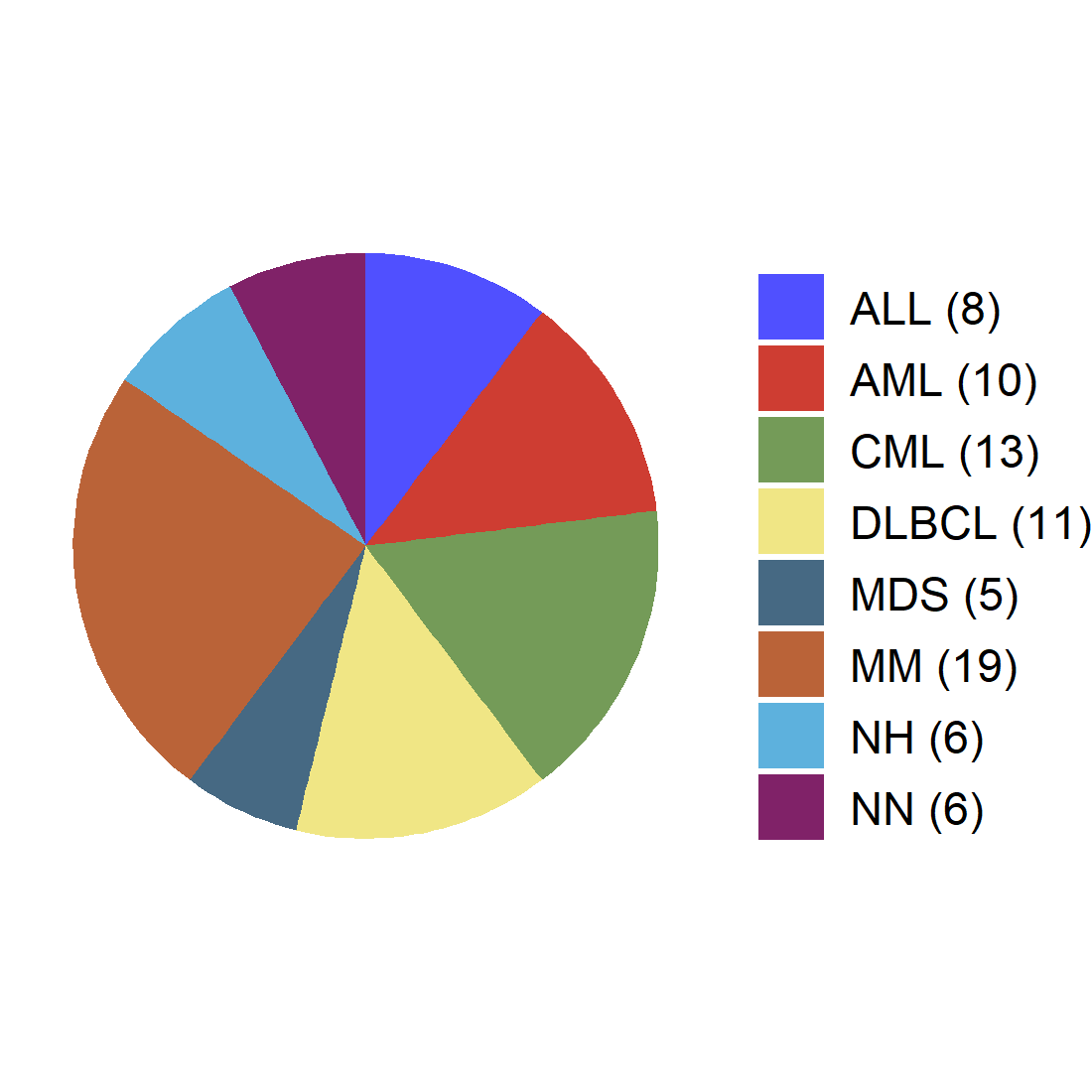

Supplement: S7 Fig — (TIFF) [file pone.0236338.s016.tiff]

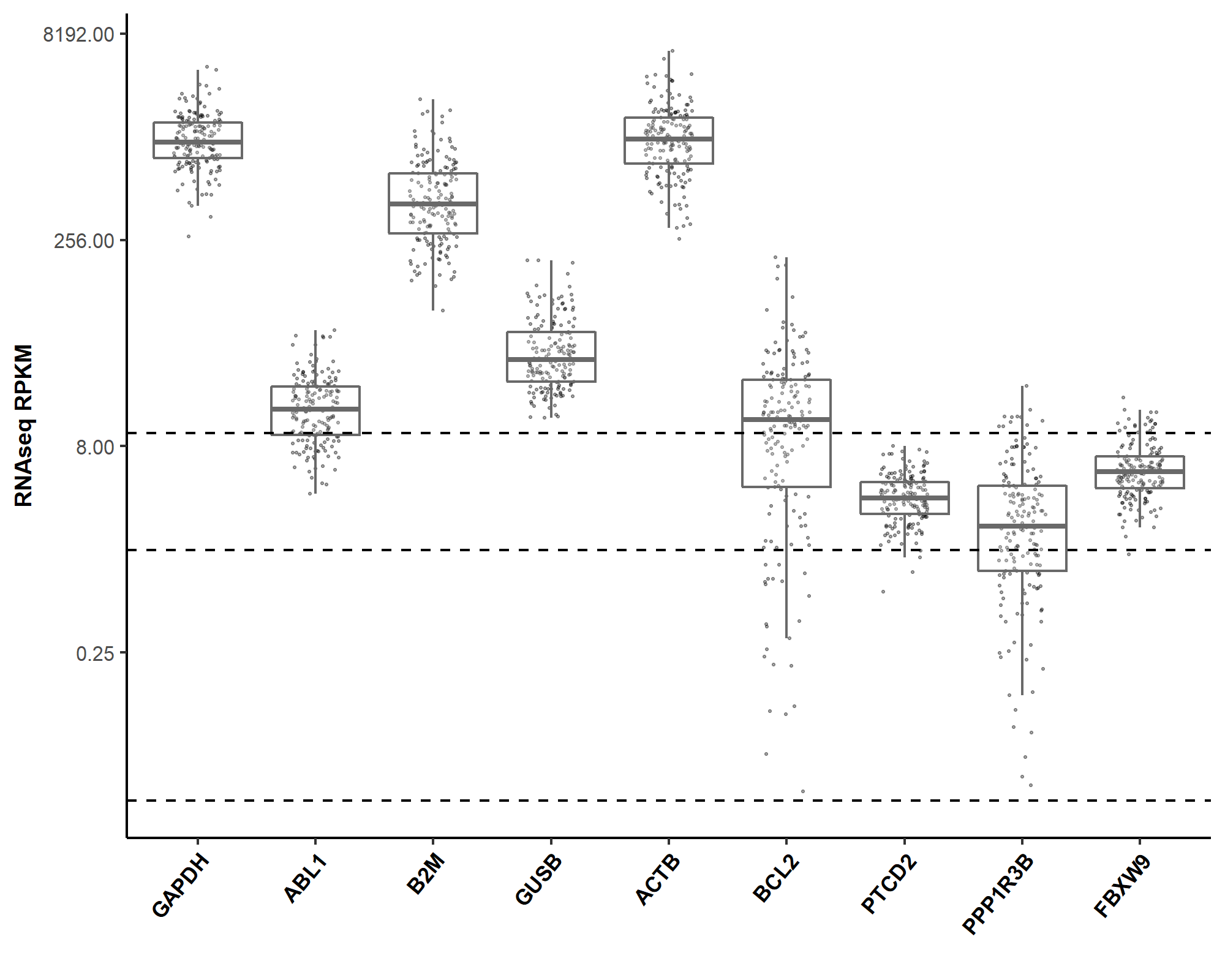

Supplement: S8 Fig — (TIFF) [file pone.0236338.s017.tiff]

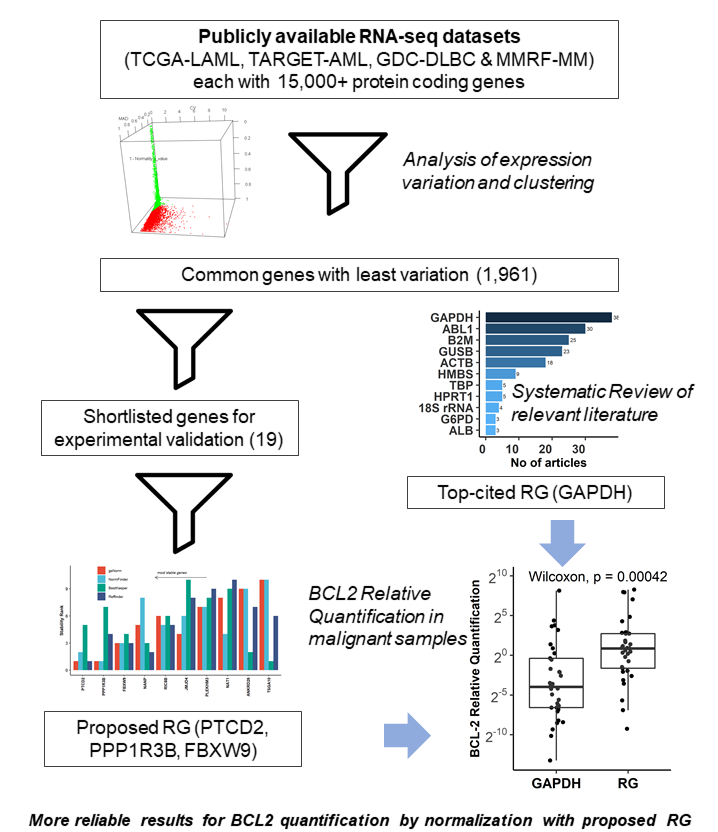

Supplement: S1 Graphical Abstract — (TIFF) [file pone.0236338.s018.tiff]
