# Supplementary material for: Relative quantification of BCL2 mRNA for diagnostic usage needs stable uncontrolled genes as reference
Source: PLoS One. 2020 Aug 12;15(8):e0236338. doi: 10.1371/journal.pone.0236338 (PMC7423076; doi:10.1371/journal.pone.0236338)
Supplement: Attachment — Submitted filename: Response to Reviewers.docx [file pone.0236338.s019.docx]

***Response to Reviewer’s comment on “Relative quantification of BCL2 mRNA for diagnostic usage needs stable uncontrolled genes as reference” by Dwivedi et al submitted to PLOS ONE***

# Responses to Reviewer 1 comments

*R1.1. Would these screened out reference genes be stable in cell lines, other than primary cells?*

**Response:** We thank the reviewer for bringing up this aspect. We checked expression of these reference genes in cell lines of haematopoietic and lymphoid tissue origin from Broad Institute Cancer Cell Encyclopaedia and found them to show lesser variations compared to common reference genes (GAPDH, ABL1, B2M, GUSB and ACTB) in cell lines as well. These results are added in revised manuscript (Results and Discussions: lines 262-268) and in supplementary data (Fig S8).

*R1.2. Have the authors compared or noticed any difference between Taqman and SyBr green in qPCR when determining the stability of reference genes?*

**Response:** We have not performed any experiment comparing stability of the reference genes between TaqMan and SYBR green probes. While performing the systematic review we came across a number of research articles [1–8] that have used TaqMan probes, but none of them present a comparison with SYBR green.

However, we came across other studies that have compared the assay using TaqMan and SYBR green probes. Cao and Shockey [9] demonstrated identical expression patterns of reference genes between SYBR Green and TaqMan qPCR for a tropical oilseed plant whereas Tajadini et al [10] measured the expression of various adenosine receptors in breast cancer tissue and found performance of the SYBR green assay as comparable with TaqMan. In a different context using human reference RNA, Arikawa et al [11] found SYBR green PCR assays having maximum concordance with TaqMan PCR.

From these evidences, we believe that stability of reference genes are not likely to differ in SYBR green qPCR assay compared to TaqMan. Hence, we have not commented on this aspect in our revised manuscript.

*R1.3.* *Would the western blot assay reflect similar results using these three stable reference genes?*

**Response:** Our study focuses on detection of BCL2 through qPCR for diagnostic purposes, hence aiming at only the mRNA expression of genes.

By design we have chosen reference genes that are of moderate expression level (in middle quartiles of expression among other genes), and they may not be detectable by Western blot unless a larger amount of sample is loaded, which is often not feasible with clinical samples.

Having said that, it may be an interesting proposition to predict stable reference proteins for use in Western blot. For this a new study can be initiated with statistical analysis of proteomics data and associated systematic review of literature.

*R1.4. How about the applicability of these three reference genes in animal model or murine cells?*

**Response:** This a very relevant comment as use of animal models, especially murine models are very common in leukemia and lymphoma research. We analyzed sequences of the proposed reference genes and found them to have 70 – 90% similarity and identity with corresponding genes in mouse or other rodent models. This result indicates that proposed reference genes may also be useful in experiments with murine models.

We have incorporated these results in revised manuscript (Results and discussion: lines 269 – 277) and in supplementary data (Table S8). We thank the reviewer for bringing out this perspective.

# Responses to Reviewer 2 Comments

R2.1. *Reference to past publications underlining the importance of a correct selection of appropriate RGs is needed.*

**Response:** We had mentioned about inconsistency in GAPDH expression as reported in literature in the Results and discussion section (lines 303 – 306). However, as suggested by reviewer we have added a paragraph on this topic (Introduction: lines 64-72) adding reference to past publications. We thank the reviewer for this valuable suggestion.

R2.2. *The authors are urged to present normalization factors with the RGs they identified and present the results.*

**Response:** Normalization factor as mentioned by reviewer refers to geometric mean of relative expression of the reference genes. We had already used this in our calculations for BCL2 relative quantitation.

However, based on reviewer’s suggestion we have explicitly mentioned the normalization factor in appropriate places of the manuscript (Materials and methods: lines 199-204, Results and discussion: lines 300-301 and Fig. 4). We have also added a table with values of normalization factors in the supplementary data (Table S9).

**References**

1.	Barbany G, Hagberg A, Olsson-Strömberg U, Simonsson B, Syvänen AC, Landegren U. Manifold-assisted reverse transcription-PCR with real-time detection for measurement of the BCR-ABL fusion transcript in chronic myeloid leukemia patients. Clin Chem [Internet]. 2000 Jul [cited 2020 Jan 10];46(7):913–20.

2.	Bolufer P, Sanz GF, Barragán E, Sanz MA, Cervera J, Lerma E, et al. Rapid quantitative detection of BCR-ABL transcripts in chronic myeloid leukemia patients by real-time reverse transcriptase polymerase-chain reaction using fluorescently labeled probes. Haematologica [Internet]. 2000 Dec 1 [cited 2020 Jun 16];85(12):1248–54.

3.	Scholl C, Breitinger H, Schlenk RF, Döhner H, Fröhling S, Döhner K. Development of a real-time RT-PCR assay for the quantification of the most frequent MLL/AF9 fusion types resulting from translocation t(9;11)(p22;q23) in acute myeloid leukemia. Genes Chromosom Cancer. 2003 Nov 1;38(3):274–80.

4.	Abruzzo L V., Lee KY, Fuller A, Silverman A, Keating MJ, Medeiros LJ, et al. Validation of oligonucleotide microarray data using microfluidic low-density arrays: A new statistical method to normalize real-time RT-PCR data. Biotechniques. 2005;38(5):785–92.

5.	Vera-Lozada G, Scholl V, Barros MHM, Sisti D, Guescini M, Stocchi V, et al. Analysis of biological and technical variability in gene expression assays from formalin-fixed paraffin-embedded classical Hodgkin lymphomas. Exp Mol Pathol. 2014 Dec 1;97(3):433–9.

6.	Lantuejoul S, Rouquette I, Blons H, Stang N Le, Ilie M, Begueret H, et al. French multicentric validation of ALK rearrangement diagnostic in 547 lung adenocarcinomas. Eur Respir J. 2015 Jul 1;46(1):207–18.

7.	Spiess B, Rinaldetti S, Naumann N, Galuschek N, Kossak-Roth U, Wuchter P, et al. Diagnostic performance of the molecular BCR-ABL1 monitoring system may impact on inclusion of CML patients in stopping trials. PLoS One. 2019 Mar 1;14(3).

8.	Casoli C, Pilotti E, Bertazzoni U. Proviral load determination of HTLV-1 and HTLV-2 in patients’ peripheral blood mononuclear cells by real-time PCR. Methods Mol Biol. 2014;1087:315–23.

9.	Cao H, Shockey JM. Comparison of TaqMan and SYBR green qPCR methods for quantitative gene expression in tung tree tissues. J Agric Food Chem. 2012 Dec 19;60(50):12296–303.

10.	Tajadini M, Panjehpour M, Javanmard S. Comparison of SYBR Green and TaqMan methods in quantitative real-time polymerase chain reaction analysis of four adenosine receptor subtypes. Adv Biomed Res. 2014;3(1):85.

11.	Arikawa E, Sun Y, Wang J, Zhou Q, Ning B, Dial SL, et al. Cross-platform comparison of SYBR® Green real-time PCR with TaqMan PCR, microarrays and other gene expression measurement technologies evaluated in the MicroArray Quality Control (MAQC) study. BMC Genomics [Internet]. 2008 Jul 11 [cited 2020 Jun 17];9(1):328. Accessed: 10.1186/1471-2164-9-328
