# Supplementary material for: Relative quantification of BCL2 mRNA for diagnostic usage needs stable uncontrolled genes as reference
Source: PLoS One. 2020 Aug 12;15(8):e0236338. doi: 10.1371/journal.pone.0236338 (PMC7423076; doi:10.1371/journal.pone.0236338)
Supplement: Attachment — Submitted filename: Response to Reviewers.docx [file pone.0236338.s020.docx]

***Response to Editor’s comments on “Relative quantification of BCL2 mRNA for diagnostic usage needs stable uncontrolled genes as reference” by Dwivedi et al submitted to PLOS ONE***

*Editor’s comments:*

Authors are requested to introduce into the manuscript two minor discussion points that were provided in the response to the Referees

1- the brief clarification on SYBR green (the discussion text in the Response letter should suffice)
2- the point on the focus on mRNA expression and referring the correlation to protein expression (as measured by Western blot) - again, the discussion text in the Response letter should suffice.

*Response:*

We thank Editor for the suggestion to introduce above discussion points in the manuscript which has further elevated the value of our work. In line with this suggestion, we have introduced a new subsection “Broader applicability of proposed reference genes” under Results and Discussion and included discussion points on SYBR green (lines 310 – 319), cell lines (lines 320 - 324), rodent models (lines 325- 333) and Western blot (lines 334 - 341) in this subsection. We think all these discussion points being under same subsection makes the flow more coherent.
